# Pt and CoB trilayer Josephson $\pi$ junctions with perpendicular magnetic anisotropy

**DOI:** 10.1038/s41598-021-90432-y

**Published:** 2021-05-27

**Authors:** N. Satchell, T. Mitchell, P. M. Shepley, E. Darwin, B. J. Hickey, G. Burnell

**Affiliations:** School of Physics and Astronomy, University of Leeds, Leeds, LS2 9JT UK

**Keywords:** Spintronics, Superconducting properties and materials, Magnetic devices, Superconducting devices, Magnetic properties and materials

## Abstract

We report on the electrical transport properties of Nb based Josephson junctions with Pt/Co$_{68}$B$_{32}$/Pt ferromagnetic barriers. The barriers exhibit perpendicular magnetic anisotropy, which has the main advantage for potential applications over magnetisation in-plane systems of not affecting the Fraunhofer response of the junction. In addition, we report that there is no magnetic dead layer at the Pt/Co$_{68}$B$_{32}$ interfaces, allowing us to study barriers with ultra-thin Co$_{68}$B$_{32}$. In the junctions, we observe that the magnitude of the critical current oscillates with increasing thickness of the Co$_{68}$B$_{32}$ strong ferromagnetic alloy layer. The oscillations are attributed to the ground state phase difference across the junctions being modified from zero to $\pi$. The multiple oscillations in the thickness range $0.2~\leqslant ~d_\text {CoB}~\leqslant ~1.4$ nm suggests that we have access to the first zero-$\pi$ and $\pi$-zero phase transitions. Our results fuel the development of low-temperature memory devices based on ferromagnetic Josephson junctions.

## Introduction

Proximity effects between superconducting (*S*) and ferromagnetic (*F*) materials are a topic of intense research effort due to the new physics at *S–F* interfaces^1–6^. In *S–F–S* Josephson junctions, it is well established that the ground-state phase difference across the junction can be tuned from zero to $\pi$, depending on the *F* layer thickness^1^. Experimentally, the zero-$\pi$ transitions correspond to oscillations in the junction’s characteristic voltage, $I_cR_N$, with increasing *F* layer thickness^7^. To date, experimental demonstrations of $\pi$-junctions include: the weak ferromagnetic alloys CuNi^8–14^, PdNi^15,16^ and PdFe^17^, the ferromagnetic elements Ni^18–26^, Co^20–22,27^ and Fe^20,21,28^, and the strong ferromagnetic alloys NiFe^20–22,29–32^, Ni$_3$Al^33^, NiFeMo^34^ and NiFeCo^31^.

**Figure 1:**
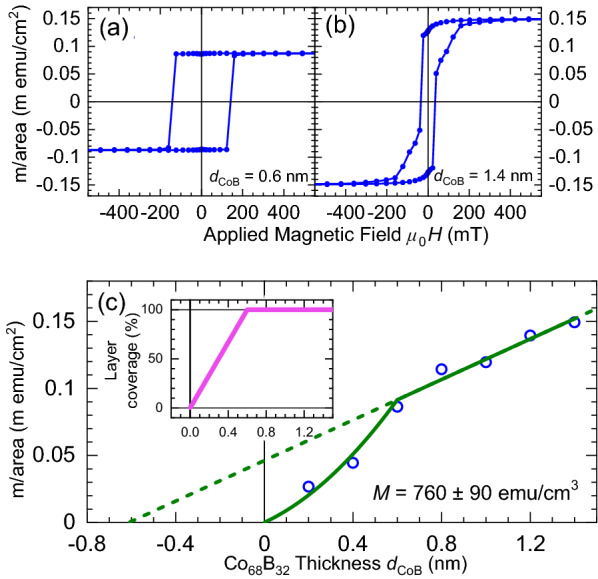
Magnetic characterisation of the sheet film samples *S*-Pt(10)-Co$_{68}$B$_{32}$(*d*$_\text {CoB}$)-Pt(5)-*S*. (**a**,**b**) Magnetic hysteresis loops of the magnetic moment (m) per area acquired at a temperature of 10 K with the applied field oriented out-of-plane for (**a**) *d*$_\text {CoB} = 0.6$ nm and (**b**) *d*$_\text {CoB} = 1.4$ nm. The diamagnetic contribution from the substrate has been subtracted. (**c**) Collated saturation moment per area versus nominal thickness of Co$_{68}$B$_{32}$. To model the lower m/area of the thinnest samples in the study, we construct a partial layer coverage model detailed in the text and shown in the inset. The result of fitting the model over the entire data range is shown by the solid line. The extracted magnetisation $M=760 \pm 90$ emu/cm$^3$. The dashed line shows an extrapolation of the linear part of the model to the intercepts. Values of m/area are calculated from the measured total magnetic moments and areas of the samples. The uncertainty in each point is dominated by the area measurements, and is less than 5%.

**Figure 2:**
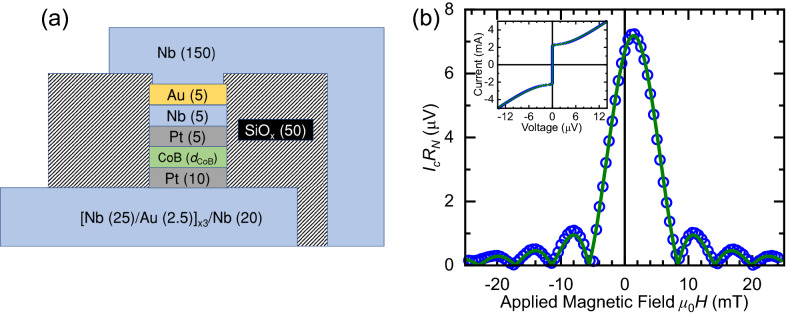
(**a**) Schematic cross section of the *S*-*F*-*S* Josephson junction device (not to scale). The thickness of each layer is given in nanometers. The Co$_{68}$B$_{32}$ layer thickness, *d*$_\text {CoB}$, is ranged from 0.2 to 1.4 nm. (**b**) Product of critical Josephson current times normal-state resistance versus applied magnetic field for the device depicted in (**a**) with *d*$_\text {CoB} = 0.6$ nm at 1.8 K. $I_c$ is determined from the measured *I*–*V* characteristic at each field value and $R_N$ is the average normal state resistance across all measured fields. The uncertainty in determining $I_cR_N$ is smaller than the data points. The data are fit with Eqs. (2) and (3). Inset: The *I*–*V* characteristic at zero applied field with fit to Eq. (1).

**Figure 3:**
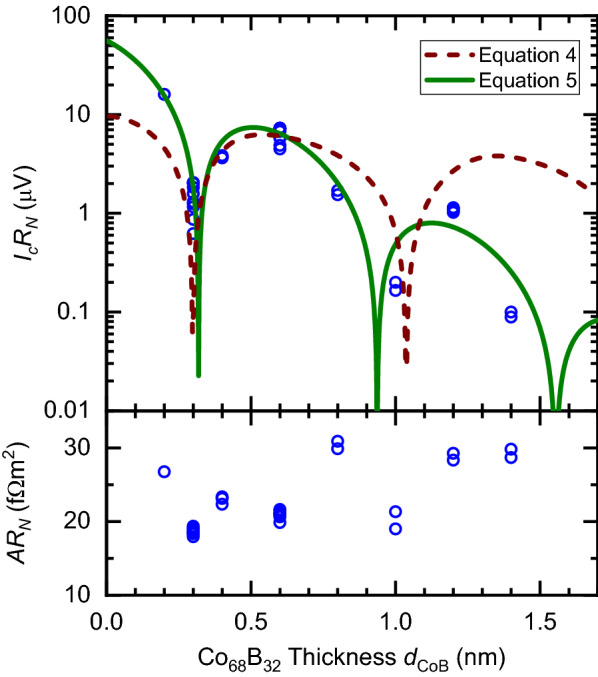
Top: Product of critical Josephson current times normal-state resistance versus nominal Co$_{68}$B$_{32}$ thickness for ferromagnetic Josephson junctions of the form *S*-Pt(10)-Co$_{68}$B$_{32}$(*d*$_\text {CoB}$)-Pt(5)-*S* at 1.8 K. Each data point represents one Josephson junction and the uncertainty in determining $I_cR_N$ is smaller than the data points. The data are fit to Eqs. (4) and (5). The best fit parameters for Eq. (4) corresponds to $\xi _{F} = 0.28\pm 0.01$ nm, and for Eq. (5) to $\xi _{F1} = 0.28\pm 0.02$ nm and $\xi _{F2} = 0.20\pm 0.02$ nm. The first minimum at $0.30\pm 0.05$ nm indicates a transition between the zero and $\pi$-phase states. Bottom: Product of the area times normal-state resistance for the same junctions. The scatter in $AR_N$ is most likely sample-to-sample variation in *A*.

**Table 1:** Best fit parameters determined for selected $\textit{S}{{-}}{} { F}{{-}}{} { S}$ Josephson junctions where the *F* layers are ferromagnetic alloys and the $I_cR_N$ oscillations are well described by Eq. (5).

*F*	$\xi _{F1}$ (nm)	$\xi _{F2}$ (nm)	$d_{\text {zero-}\pi }$ (nm)	$V_0$	References
				($$\mu $$V)	
Pd$_{97}$Fe$_3$	$16.2\pm 1.4$	$7.2\pm 0.6$	$16.3 \pm 0.2$	$102 \pm 12$	^17^
Ni$_{80}$Fe$_{20}$	$1.50 \pm 0.38$	$0.58 \pm 0.10$	$1.76 \pm 0.05$	$69 \pm 19$	^31^
Ni$_{65}$Fe$_{15}$Co$_{20}$	$1.11 \pm 0.16$	$0.48 \pm 0.03$	$1.15 \pm 0.02$	$30 \pm 6$	^31^
Ni$_{73}$Fe$_{21}$Mo$_6$	$0.48 \pm 0.04$	$0.955 \pm 0.004$	$2.25 \pm 0.10$	$150 \pm 50$	^34^
Co$_{68}$B$_{32}$	$0.28 \pm 0.02$	$0.20 \pm 0.02$	$0.30\pm 0.05$	$56 \pm 8$	This work

In general, most previous works measure Josephson junctions with *F* layers having in-plane magnetisation. When the magnetisation is in-plane, the *F* layer can contribute significant magnetic flux density in the junction, modifying the response of the junction to an externally applied measurement field and shifting the maximum critical current away from $H=0$. In addition, when applying an in-plane field to measure the junction, the *F* layer may switch in the measurement field. Josephson junctions containing perpendicular magnetic anisotropy (PMA) *F* layers have advantages over in-plane systems as, in principle, the magnetisation and magnetic switching of layers in the junction should not affect the in-plane magnetic flux. The application of an in-plane measurement field will tilt the magnetisation of the PMA layer slightly, however the field required to fully saturate the PMA layers considered in this work is far larger than the field required to characterise the junctions, so the tilting effect can be neglected.

Of the previous *F* layers characterised, only CuNi and PdNi have an intrinsic PMA component of their magnetisation. An alternative to intrinsic PMA is interfacial PMA, which can give a *F* layer an overall PMA so long as the *F* layer is thin enough that the interfacial anisotropy dominates over the bulk anisotropy. Josephson junctions containing interfacial PMA *F* layers have been previously studied theoretically^35–37^ and experimentally in the context of spin-triplet supercurrents, however, no zero-$\pi$ oscillations were expected or observed in the particular geometries studied^38–41^.

In this work, we study the amorphous strong ferromagnetic alloy Co$_{68}$B$_{32}$^42^. For many spintronic applications, the amorphous Co based alloys are advantageous over crystalline Co due to their lack of crystalline anisotropy and weaker pinning of magnetic domain walls due to the reduced density of grain boundaries^43^. Recently, thin film Co$_{68}$B$_{32}$ has been studied for magnetic memory application and as a host of magnetic skyrmions^44–46^. When placed adjacent to Pt layers, the Pt/Co$_{68}$B$_{32}$ interfaces exhibit PMA, giving an overall PMA for the thin layers considered in this work. Previously, we used Co$_{68}$B$_{32}$ in PMA pseudospin-valve junctions, where the critical current of the junction could be controlled by the relative orientation of two ferromagnets in the Pt/Co/Pt/Co$_{68}$B$_{32}$/Pt barrier^47^. For application in cryogenic memory, it is important to demonstrate that in addition to modulating the critical current of such devices, it is also possible to switch such devices from the zero to $\pi$ state^48–51^. For this, the zero–$\pi$ critical current oscillations of the component ferromagnets in the pseudospin-valve should be well characterised. In this work, we present evidence of such zero–$\pi$ critical current oscillations in Pt/Co$_{68}$B$_{32}$/Pt junctions.

## Results

### Magnetic characterisation

Magnetic moment per area versus out-of-plane field data are shown in Fig. 1a,b for *S*-Pt(10)-Co$_{68}$B$_{32}$(*d*$_\text {CoB}$)-Pt(5)-*S* sheet film samples at 10 K with a nominal thickness (a) *d*$_\text {CoB} = 0.6$ nm and (b) *d*$_\text {CoB} = 1.4$ nm. For *d*$_\text {CoB} = 0.6$ nm, the square hysteresis loop indicates a strong PMA. As the nominal thickness of the Co$_{68}$B$_{32}$ is increased towards the largest thickness studied in this work, *d*$_\text {CoB} = 1.4$ nm Fig. 1b, we observe two changing characteristics in the hysteresis loops. Firstly, the coercive field reduces. Secondly, the squareness ratio of the loop reduces - indicating competing anisotropies in the Co$_{68}$B$_{32}$ layer. Upon making the CoB thicker, we would expect that the anisotropy of the layer will change from being predominately PMA to predominately in-plane.

Saturation magnetic moment per area versus nominal thickness of the Co$_{68}$B$_{32}$(*d*$_\text {CoB}$) at 10 K are shown in Fig. 1c. These data are best described in two regimes. For the thicker samples in this study, we observe the expected linear dependence with increasing thickness of ferromagnet. The thinnest two samples in this study deviate from this linear trend, showing a lower moment/area than implied by the trend in the thicker samples.

In order to apply a fitting model which describes the entire data set, we construct a partial layer coverage toy model of our system. The basis of the model is the assumption that the *F*-layers in the thinnest samples may not be continuous. From zero thickness to some critical thickness, we assume that the layer coverage increases linearly by the profile shown in Fig. 1c inset. The physical picture implied by this model is consistent with island nucleation, coalescence followed by layered growth – which is not an atypical growth mode for ambient temperature sputtered thin films. Above the critical thickness, we assume that the layer is now continuous, so the data can be described by the expected linear trend. More information on the model and extracting magnetisation from moment/area data are included in the Supplementary Information along with alternative fitting models to the data presented in Fig. 1c (see Supplementary Fig. S1 online).

The result of our toy model is shown by the solid line in Fig. 1c. The toy model gives a critical thickness for layer growth of $0.6\pm 0.1$ nm, which is comparable to a couple of unit cells of nominal thickness, and gives the magnetisation of the Co$_{68}$B$_{32}$ to be $M=760\pm 90$ emu/cm$^3$, consistent with the expected bulk magnetisation of 730 emu/cm$^3$^52^. The model also includes a contribution to the total magnetic signal from the polarisation of the adjacent Pt layer, this is commonly observed in such systems^53–57^. In Figure 1 (c), the polarised Pt contribution can be extracted from the y-intercept of the dashed line, which is an extrapolation of the linear part of the model. The magnetic contribution to the total magnetic response of the sample by the polarised Pt is $46 \pm 9~\mu$emu/cm$^2$, or $23 \pm 5~\mu$emu/cm$^2$ per Pt/Co$_{68}$B$_{32}$ interface, consistent with Suzuki *et al.*^55^.

It has been reported elsewhere that significant magnetic dead layers can form in ferromagnetic Josephson junction barriers at the Nb/*F* interfaces, see for example^21^, however adding buffer layers such as Rh, Cu or Pt can significantly improve the morphology of the *F* layer^27,41,58,59^. The signature of magnetic dead layers is a positive x-intercept when plotting moment/area versus thickness. In our Pt/Co$_{68}$B$_{32}$/Pt barriers, the x-intercept when fitting to Fig. 1c is not positive (regardless of the model used, see Supplementary Fig. S1 online), suggesting that such dead layers have been minimised by the Pt interfaces. Additionally, when the nominal thickness of the Co$_{68}$B$_{32}$ is equivalent to only one or two monolayers, and so the layer is modelled with partial coverage, the polarised Pt appears to have stabilised the magnetisation of what we expect are islands of Co$_{68}$B$_{32}$, allowing us to measure a magnetic response even for *d*$_\text {CoB} = 0.2$ nm, a significant advantage of our approach.

### Electrical transport

Samples were fabricated using standard lithography techniques into circular current perpendicular-to-plane Josephson junction devices, as depicted schematically in Fig. 2a. We load the devices into our cryostat at room temperature and first cool to 15 K, just above the superconducting transition (9 K), where we apply a 1 T out-of-plane saturating field. Once the saturating field is removed, we rotate the sample so the field is applied in-plane and cool the samples to the base temperature of our cryostat, 1.8 K. We measure the *I–V* characteristic of each junction as a function of in-plane applied magnetic field between $\pm 25$ mT to determine the Fraunhofer pattern. The field necessary to saturate our Pt/Co$_{68}$B$_{32}$/Pt barriers is in excess of 1 T so the maximum deviation of the magnetisation from the perpendicular is less than 1.5$^\circ$.

The *I–V* characteristics of our devices follow the standard square-root form expected for over-damped Josephson junctions^60^,

1 $$\begin{aligned} V=R_N\sqrt{I^2 - I^2_{c}},~\text {for}~I \ge I_{c} \end{aligned}$$

where $I_c$ is the critical Josephson current and $R_N$ is the normal state resistance of the junction. For circular Josephson junctions, the $I_c$(*B*) Fraunhofer response can be described by the Airy function^60^,

2 $$\begin{aligned} I_c = I_{c0} \left| 2J_1 (\pi \Phi / \Phi _0)/(\pi \Phi / \Phi _0) \right| , \end{aligned}$$

where $I_{c0}$ is the maximum critical current, $J_1$ is a Bessel function of the first kind, $\Phi _0=h/2e$ is the flux quantum, and $\Phi$ is the flux through the junction^60^,

3 $$\begin{aligned} \Phi = \mu _0 (H_\text {app} - H_\text {shift}) w \big [ \lambda ^\text {bottom}_\text {L} \tanh {(d^\text {bottom}_S/2\lambda ^\text {bottom}_\text {L})} + \lambda ^\text {top}_\text {L} \tanh {(d^\text {top}_S/2\lambda ^\text {top}_\text {L})} + d \big ], \end{aligned}$$

where *w*, $\lambda _\text {L}$, $d_S$, and *d* are the width of the junction, the London penetration depth, the thickness of the superconducting electrode, and the total thickness of all the normal metal layers and *F* layers in the junction, respectively. The bottom electrode is a Nb/Au multilayer ($\lambda ^\text {bottom}_\text {L}$ = 190 nm^61^) and the top electrode is single layer Nb ($\lambda ^\text {top}_\text {L}$ = 150 nm^62^). $H_\text {app}$ is the applied field and $H_\text {shift}$ is the amount $I_{c0}$ is shifted from *H* = 0. $H_\text {shift}$ arises from a combination of an intrinsic contribution due to any in-plane magnetisation of the junction, and extrinsic artifacts from trapped flux in the 3$\,\mathrm {T}$ superconducting coil used to perform the measurements. Fits to these equations are shown along with the data on a typical device in Fig. 2b. We attribute the small $H_\text {shift}$ in Fig. 2b to trapped flux in our superconducting coil, which is supported by additional Fraunhofer data for both +1 T and -1 T saturation fields (see Supplementary Fig. S2 online). We determine $I_{c0}$ for many samples of different Co$_{68}$B$_{32}$ thicknesses (*d*$_\text {CoB}$) following the same protocol.

Figure 3 shows the collated $I_cR_N$ and $AR_N$ (area times normal-state resistance) products for the Josephson junctions measured in this study. $I_c$ corresponds to the $I_{c0}$ maximum of the $I_c$(*B*) Fraunhofer response and $R_N$ is the average resistance from measurements at all field values. As the thickness of the Co$_{68}$B$_{32}$ is increased, the $I_cR_N$ shows nonmonotonic behaviour. When plotting $AR_N$, we fix *A* by the nominal design dimension. The $AR_N$ product for our samples is suggestive that within the same chip the junction-to-junction reproducibility is very good, which is also supported by the small spread of $I_cR_N$ values for junctions on the same chip. It is possible to determine *A* by fitting $I_c$(*B*) to Eqs. (2) and (3), and we find that across all our junctions the extracted average $\bar{w} = 3.0\pm 0.3~\mu$m is consistent with the lithography design. The scatter in $AR_N$ is therefore similar to the scatter in the linear dimensions of the junctions. Variations in *A* between samples will not affect the reported $I_cR_N$, which is a size independent quantity. Indeed, there is no correlation between a high/low $I_cR_N$ and $AR_N$.

We report strong reproduciblity of our results, as multiple samples for $d_\text {CoB}= 0.3$ and 0.6 nm are grown and fabricated in independent cycles and show consistency in $I_cR_N$, Fig. 3. Scatter in $I_cR_N$ is most likely driven by sample-to-sample variations in the thickness of the Co$_{68}$B$_{32}$.

### Coherence lengths in *S*/*F*/*S* Josephson junctions

The transport properties of *S–F–S* Josephson junctions are well described in three limits, driven by the relative magnitude of three lengthscales; the mean free path ($l_e$), the superconducting coherence length ($\xi _S$) and the effective coherence length inside the ferromagnet ($\xi _F$). In the ballistic limit $l_e> \xi _S > \xi _F$, in the intermediate limit $\xi _S> l_e > \xi _F$, and in the diffusive limit $\xi _S> \xi _F > l_e$.

In the ballistic limit, the decay and oscillations of $I_cR_N$ is given by the numerical maximum with respect to $\varphi$ of the ballistic limit supercurrent $I_\text {S}(\varphi )$^7^,

4 $$\begin{aligned} I_\text {S}(\varphi )R_N = \frac{\pi \Delta \alpha ^2}{2 e} \int _{\alpha }^{\infty }\frac{dy}{y^3}\Bigg ( \sin {}\frac{\varphi -y}{2}\tanh {}\frac{\Delta \cos {}\frac{\varphi -y}{2}}{2k_\text {B}T} + \sin {}\frac{\varphi +y}{2}\tanh {}\frac{\Delta \cos {}\frac{\varphi +y}{2}}{2k_\text {B}T}\Bigg ), \end{aligned}$$

where $\varphi$ is the phase difference across the junction, $\Delta$ is the energy gap, *T* is the temperature, and $\alpha \equiv d / \xi _F$. In the ballistic limit, $\xi _F =\hbar v_F /2E_\text {Ex}$, where $v_F$ is the Fermi velocity and $E_\text {Ex}$ is the exchange energy. Ballistic limit transport has been reported in the ferromagnetic elements when sufficiently thin, for example in Ni barriers studied by Robinson *et al.*^20^ and Baek *et al.*^25^.

In the intermediate limit, the decay and oscillations of $I_cR_N$ is given by^63^,

5 $$\begin{aligned} I_cR_N = V_0 \exp \bigg ( \frac{-d_F}{\xi _{F1}} \bigg ) \bigg |\sin \bigg ( \frac{d_F - d_{\text {zero--}\pi }}{\xi _{F2}} \bigg )\bigg |, \end{aligned}$$

where $d_{\text {zero--}\pi }$ is the thickness of the first zero–$\pi$ transition, $\xi _{F1} = l_e$ and $\xi _{F2}=\xi _{F}$ are the lengthscales governing the decay and oscillation of $I_cR_N$, respectively. In the intermediate limit, one finds $\xi _{F1} > \xi _{F2}$. Most ferromagnetic alloys studied in ferromagnetic Josephson junctions are solid-solutions with short mean free paths and are found to be best described in the intermediate limit^17,31^, for example PdNi barriers studied by Khaire *et al.*^16^.

It is well established that Rashba effects arising from spin-orbit coupling can be found at the interface between metallic ferromagnets, such as Co, and heavy metals, such as Pt^64–66^ and so in the diffusive limit with spin-flip or spin-orbit scattering, the transport may be described by Eq. (5)^67^. However in the diffusive limit one will find $\xi _{F2} > \xi _{F1}$^67,68^. In experimental literature, this situation is somewhat rarer, for example CuNi barriers studied by Oboznov *et al.*^10^ and NiFeMo barriers studied by Niedzielski *et al.*^34^.

Fitting to our results taking $\xi _{F}$ and $\xi _{F1,2}$ as fitting parameters (shown in Fig. 3), we find that Eq. (4) does not reproduce our Co$_{68}$B$_{32}$ data as well as Eq. (5), particularly for larger $d_\text {CoB}$. The fits for Eq. (5) correspond to the limit $\xi _{F1} > \xi _{F2}$, placing our junctions in the intermediate limit. The best fit parameters are given in Table 1, which also includes results from other ferromagnetic alloys best described by Eq. (5). For further analysis on our data see Supplementary Fig. S3 and S4 online.

## Discussion

Systems, such as ours, with a source of *s*-wave superconductivity, large spin-orbit coupling, and ferromagnetism are predicted to display transport properties consistent with spin-triplet supercurrents^69^, reported to be observed in ferromagnetic resonance measurements^70^. In this work, however, within the resolution of our measurements, the data are well described by singlet transport physics alone and we do not need to invoke a significant spin-triplet supercurrent to explain our results – for further details see the Supplementary Information online. The lack of evidence for spin-triplet supercurrents in these Josephson junctions is consistent with previous works^41,59^.

In comparison to other ferromagnetic alloys, such as those in Table 1, our Co$_{68}$B$_{32}$ junctions display significantly shorter $\xi _{F1}$ and $\xi _{F2}$ characteristic lengthscales. We attribute the short $\xi _{F1}$ to the common property of amorphous alloys having a short $l_e$ due to structural disorder, however scattering at the Pt/Co$_{68}$B$_{32}$ interfaces may also be considerable for very thin *F* layers. The advantage of the short $\xi _{F2}$ in our system is that we have access to the first zero-$\pi$ and $\pi$-zero transitions before the *F* layer undergoes the reorientation transition to in-plane magnetisation. Despite the short $\xi _{F1}$, the extrapolated $I_cR_N$ at zero thickness for our junctions, $V_0 = 56\pm 8~\mu$V, is comparable to Ni$_{80}$Fe$_{20}$ junctions, $V_0 = 69\pm 19~\mu$V^31^, which have been extensively studied for similar applications^20–22,29–32,48–51^. In addition, the $I_cR_N$ product at the peak of the first $\pi$ state, $V_\pi \approx 7 \mu$V, is comparable to other ferromagnetic alloys, for example $V_\pi \approx 5 \mu$V in Ni$_{65}$Fe$_{15}$Co$_{20}$ and $V_\pi \approx 12 \mu$V in Ni$_{80}$Fe$_{20}$^31^. The disadvantage of the short $\xi _{F2}$ for application is that precise control over thickness is necessary, since small variations will cause large changes to the $I_cR_N$ product and could potentially change the phase difference across the junction. Fortunately, such PMA multilayer stacks have an established industrial process for applications in magnetic recording.

The key application of ferromagnetic Josephson junctions is Josephson magnetic RAM (JMRAM)^71^. In this technology, the Co$_{68}$B$_{32}$ forms one layer in a pseudospin-valve Josephson junction of the general type reported in our previous publication where we used Co and Co$_{68}$B$_{32}$^47^. In order to use the pseudospin-valve for JMRAM, the component ferromagnetic layers must be well characterised, as we report here for Pt/Co$_{68}$B$_{32}$/Pt PMA trilayers. In JMRAM, the zero-$\pi$ ferromagnetic junction is a passive phase shifter in a SQUID loop containing two *S*/*I*/*S* junctions. As the zero-$\pi$ junction is passive, there is no need for this junction to have a large $I_cR_N$, however as we demonstrate our Co$_{68}$B$_{32}$ has comparable performance in this regard to Ni$_{80}$Fe$_{20}$, which is used in state-of-the-art devices. A JMRAM memory cell with in-plane ferromagnets (Ni$_{80}$Fe$_{20}$ and Ni) was recently demonstrated by Dayton *et al.*^50^.

The detrimental features of magnetisation in-plane junctions are driven by the interaction between the physics underlying the Fraunhofer response and the vector potential of the ferromagnet. For in-plane single domain junctions, the Fraunhofer pattern will be uniformly shifted from zero global applied field by the vector potential of the ferromagnet in the plane of the junction. For example, Glick *et al.* characterise candidate in-plane *F* layers^31^, where the Fraunhofer patterns are uniformly shifted by $H_\text {shift}\propto -Md_F$. To ensure that the *F* layers are single domain, the maximum junction area in that work is $0.5\mu$m$^2$. If the area of the junctions is increased so that the ferromagnet becomes multidomain, it may not be possible to recover a Fraunhofer pattern due to the distortion by the stray fields emanating from the domains^16,58,72^. Such size considerations place an upper limit on the critical current of devices. Finally, the in-plane measurement field required to characterise the Fraunhofer pattern can cause premature switching of in-plane ferromagnets. Combined, the shift and premature switching means that JMRAM devices with in-plane ferromagnets may not have access to the highest critical current state, as observed for in-plane pseudospin-valve devices by Niedzielski *et al.*^32^.

In contrast, the use of PMA ferromagnets as we have demonstrated offers solutions to the issues introduced by in-plane ferromagnets. As the direction of the magnetisation and stray fields of PMA layers are parallel to the direction of current in the junction, they do not shift or distort the Fraunhofer pattern. Furthermore, due to the favourable demagnetisation effects, PMA materials systems have less stray fields and larger domain size limits compared to in-plane systems. Combined, these advantages allow us to successfully measure well defined Fraunhofer patterns on junctions with an area of $\approx 7\mu$m$^2$, much larger than those Glick *et al.* require to characterise in-plane layers. As a result of the PMA, the highest critical current state of the junction is available at zero global applied field, as demonstrated in Fig. 2b. Finally, the application of in-plane measurement fields will not switch a PMA ferromagnet.

For practicable memory devices, it will be important to remove the need for an applied out-of-plane switching field. Such a field to switch a PMA ferromagnet may introduce flux vortices into the superconducting Nb electrodes, which we negated in this work by applying such fields only above $T_c$. Routes towards removing this restriction by implementing an all electrical switching process include spin-transfer torque and spin-orbit torque switching of the barriers^65,66,73^.

## Conclusions

In conclusion, we demonstrate Josephson $\pi$-junctions with Pt/Co$_{68}$B$_{32}$/Pt perpendicular magnetic anisotropy barriers. Co$_{68}$B$_{32}$ is a strong ferromagnetic amorphous alloy of interest in spintronics due to its low pinning properties. We show that at the Pt/Co$_{68}$B$_{32}$ interfaces there is significant polarisation of the Pt and that the samples are magnetic down to a nominal Co$_{68}$B$_{32}$ thickness of 0.2 nm. In Josephson junctions, as the thickness of Co$_{68}$B$_{32}$ is increased, we observe the nonmonotonic decay and oscillation of the critical Josephson current. These oscillations are attributed to the junctions undergoing the zero to $\pi$ transition. $\pi$-junctions have important applications in superconducting electronics, including cryogenic memory. Systematic material studies are crucial for the development of such technologies. The performance of our perpendicular magnetic anisotropy $\pi$-junctions are at least comparable to that of NiFe, which has in-plane magnetisation.

## Methods

Samples are deposited, fabricated, and measured using identical methodology to our previous work^47^. The final product of cleanroom processing are standard “sandwich” planar Josephson junctions, defined by photolithography and Ar$^+$ ion milling, where the current flows perpendicular to the plane. The diameter of the circular junctions is a design parameter and is nominally $3~\mu$m.

We dc sputter deposit the multilayer samples onto thermally oxidised Si substrates in the Royce Deposition System^74^. The magnetrons are mounted below, and confocal to, the substrate with source-substrate distances of 134 mm. The base pressure of the vacuum chamber is 1$\times$10$^{-9}\,\mathrm {mbar}$. The samples are deposited at room temperature with an Ar (6N purity) gas pressure of 3.6$\times$10$^{-3}\,\mathrm {mbar}$ for the [Nb/Au]$_\text {x3}$/Nb bottom electrode layers and 4.8$\times$10$^{-3}\,\mathrm {mbar}$ for the Pt/Co$_{68}$B$_{32}$/Pt barrier layers. The [Nb/Au]$_\text {x3}$/Nb superlattice is used for the bottom electrode as the superlattice has a lower surface roughness compared to a single Nb layer of comparable total thickness^61,75^. Finally, a Nb/Au cap is deposited to prevent oxidation during the processing. In the final stage of sample fabrication, the top electrode, 150 nm of Nb, is deposited after an *in-situ* ion milling process to remove 5 nm from the 10 nm Au cap. The full structure of the final device with thickness in (nm) is [Nb (25)/Au(2.5)]$_\text {x3}$/Nb (20)/Pt (10)/Co$_{68}$B$_{32}$ (*d*$_\text {CoB}$)/ Pt (5)/Nb (5)/Au (5)/ Nb (150).

The choice of Pt thicknesses in this study is informed by previous results^41^, being a balance between developing a suitable textured surface on which to grow the Co$_{68}$B$_{32}$ layer whilst not being in a range that significantly affects the critical current density. The total thickness of the Pt in this work is the same as that required for pseudospin-valve devices^47^.

Fabricated devices are measured in a continuous flow $^4\mathrm {He}$ cryostat with 3$\,\mathrm {T}$ horizontal superconducting Helmholtz coils. The sample can be rotated about the vertical axis, which we perform in increments of $90^{\circ }$ to bring that field in- and out-of-plane of the junctions. In order to avoid trapping flux in the superconducting Nb layers in the devices, we performed all sample rotations in zero field above the $T_c$ of the Nb and always cooled the sample in zero applied field (in practice there will inevitably be a small remanent field due to trapped flux in the magnet). The full sequence of setting the magnetic state of our samples and performing the measurement field sweeps is: warm to 15K, rotate sample to apply field out-of-plane, apply saturating field, remove saturating field, rotate sample by $90^{\circ }$, cool sample, apply field in-plane, measure. Once we have finished measuring that magnetic state of the sample, we may wish to measure a further condition, such as reversing the magnetisation. To do so, we remove the in-plane field, warm to 15K and repeat the cycle described above.

Traditional 4-point-probe transport geometry is used to measure the current-voltage characteristic of the junction with combined Keithley 6221-2182A current source and nano-voltmeter. Magnetisation loops of sheet films are measured using a Quantum Design MPMS 3 magnetometer.

## Supplementary Information

Supplementary Information.

## Data Availability

The datasets generated during the current study are available in the University of Leeds repository, https://doi.org/10.5518/817.

## References

[1] Buzdin AI (2005). Proximity effects in superconductor-ferromagnet heterostructures. Rev. Mod. Phys..

[2] Bergeret FS, Volkov AF, Efetov KB (2005). Odd triplet superconductivity and related phenomena in superconductor-ferromagnet structures. Rev. Mod. Phys..

[3] Eschrig M (2011). Spin-polarized supercurrents for spintronics. Phys. Today.

[4] Linder J, Robinson JWA (2015). Superconducting spintronics. Nat. Phys..

[5] Eschrig M (2015). Spin-polarized supercurrents for spintronics: a review of current progress. Rep. Prog. Phys..

[6] Birge NO (2018). Spin-triplet supercurrents in Josephson junctions containing strong ferromagnetic materials. Philos. Trans. R. Soc. A.

[7] Buzdin AI, Bulaevskii LN, Panyukov SV (1982). Critical-current oscillations as a function of the exchange field and thickness of the ferromagnetic metal (F) in an SFS Josephson junction. JETP Lett..

[8] Ryazanov VV (2001). Coupling of Two Superconductors through a Ferromagnet: evidence for a $${\pi }$$ Junction. Phys. Rev. Lett..

[9] Sellier H, Baraduc C, Lefloch F, Calemczuk R (2003). Temperature-induced crossover between $$0$$ and $${\pi }$$ states in S/F/S junctions. Phys. Rev. B.

[10] Oboznov VA, Bol’ginov VV, Feofanov AK, Ryazanov VV, Buzdin AI (2006). Thickness dependence of the Josephson ground states of superconductor-ferromagnet-superconductor junctions. Phys. Rev. Lett..

[11] Weides M (2006). $$0{\text{-}}\pi $$ Josephson tunnel junctions with ferromagnetic barrier. Phys. Rev. Lett..

[12] Weides M (2006). High quality ferromagnetic 0 and $$\pi $$ Josephson tunnel junctions. Appl. Phys. Lett..

[13] Stoutimore MJA (2018). Second-harmonic current-phase relation in Josephson junctions with ferromagnetic barriers. Phys. Rev. Lett..

[14] Bolginov VV, Rossolenko AN, Shkarin AB, Oboznov VA, Ryazanov VV (2018). Fabrication of Optimized superconducting phase inverters based on superconductor-ferromagnet-superconductor $$\pi $$-junctions. J. Low Temp. Phys..

[15] Kontos T (2002). Josephson junction through a thin ferromagnetic layer: negative coupling. Phys. Rev. Lett..

[16] Khaire TS, Pratt WP, Birge NO (2009). Critical current behavior in Josephson junctions with the weak ferromagnet PdNi. Phys. Rev. B.

[17] Glick JA, Loloee R, Pratt WP, Birge NO (2017). Critical current oscillations of Josephson junctions containing PdFe nanomagnets. IEEE Trans. Appl. Supercond..

[18] Blum Y, Tsukernik A, Karpovski M, Palevski A (2002). Oscillations of the superconducting critical current in Nb-Cu-Ni-Cu-Nb junctions. Phys. Rev. Lett..

[19] Shelukhin V (2006). Observation of periodic $${\pi }$$-phase shifts in ferromagnet-superconductor multilayers. Phys. Rev. B.

[20] Robinson JWA, Piano S, Burnell G, Bell C, Blamire MG (2006). Critical current oscillations in strong ferromagnetic $${\pi }$$ junctions. Phys. Rev. Lett..

[21] Robinson JWA, Piano S, Burnell G, Bell C, Blamire MG (2007). Zero to $${\pi }$$ transition in superconductor-ferromagnet-superconductor junctions. Phys. Rev. B.

[22] Robinson JWA, Piano S, Burnell G, Bell C, Blamire MG (2007). Transport and magnetic properties of strong ferromagnetic pi-junctions. IEEE Trans. Appl. Supercond..

[23] Bannykh AA (2009). Josephson tunnel junctions with a strong ferromagnetic interlayer. Phys. Rev. B.

[24] Baek B, Rippard WH, Benz SP, Russek SE, Dresselhaus PD (2014). Hybrid superconducting-magnetic memory device using competing order parameters. Nat. Commun..

[25] Baek B, Schneider ML, Pufall MR, Rippard WH (2017). Phase offsets in the critical-current oscillations of Josephson junctions based on Ni and Ni-($$\text{Ni }_{81}\text{ Fe}_{19})_{x}\text{ Nb}_{y}$$ barriers. Phys. Rev. Appl..

[26] Baek B, Schneider ML, Pufall MR, Rippard WH (2018). Anomalous supercurrent modulation in Josephson junctions with Ni-based barriers. IEEE Trans. Appl. Supercond..

[27] Robinson JWA, Barber ZH, Blamire MG (2009). Strong ferromagnetic Josephson devices with optimized magnetism. Appl. Phys. Lett..

[28] Piano S, Robinson JWA, Burnell G, Blamire MG (2007). 0-$$\pi $$ oscillations in nanostructured Nb/Fe/Nb Josephson junctions. Eur. Phys. J. B.

[29] Bell C, Loloee R, Burnell G, Blamire MG (2005). Characteristics of strong ferromagnetic Josephson junctions with epitaxial barriers. Phys. Rev. B.

[30] Abd El Qader M (2014). Switching at small magnetic fields in Josephson junctions fabricated with ferromagnetic barrier layers. Appl. Phys. Lett..

[31] Glick JA (2017). Critical current oscillations of elliptical Josephson junctions with single-domain ferromagnetic layers. J. Appl. Phys..

[32] Niedzielski BM (2018). Spin-valve Josephson junctions for cryogenic memory. Phys. Rev. B.

[33] Born F (2006). Multiple $$0\text{- }\pi $$ transitions in superconductor/insulator/ferromagnet/superconductor Josephson tunnel junctions. Phys. Rev. B.

[34] Niedzielski BM, Gingrich EC, Loloee R, Pratt WP, Birge NO (2015). S/F/S Josephson junctions with single-domain ferromagnets for memory applications. Supercond. Sci. Technol..

[35] Margaris I, Paltoglou V, Flytzanis N (2010). Zero phase difference supercurrent in ferromagnetic Josephson junctions. J. Phys. Condens. Matter.

[36] Halterman K, Valls OT, Wu C-T (2015). Charge and spin currents in ferromagnetic Josephson junctions. Phys. Rev. B.

[37] Silaev MA, Tokatly IV, Bergeret FS (2017). Anomalous current in diffusive ferromagnetic Josephson junctions. Phys. Rev. B.

[38] Gingrich EC (2012). Spin-triplet supercurrent in Co/Ni multilayer Josephson junctions with perpendicular anisotropy. Phys. Rev. B.

[39] Glick JA (2017). Spin-triplet supercurrent in Josephson junctions containing a synthetic antiferromagnet with perpendicular magnetic anisotropy. Phys. Rev. B.

[40] Glick JA (2018). Phase control in a spin-triplet SQUID. Sci. Adv..

[41] Satchell N, Loloee R, Birge NO (2019). Supercurrent in ferromagnetic Josephson junctions with heavy-metal interlayers. II. Canted magnetization. Phys. Rev. B.

[42] Tanaka H (1993). Electronic structure and magnetism of amorphous Co$$_{1-x}$$B$$_{x}$$ alloys. Phys. Rev. B.

[43] Lavrijsen R (2010). Reduced domain wall pinning in ultrathin Pt/Co$$_{100-x}$$B$$_x$$/Pt with perpendicular magnetic anisotropy.. Appl. Phys. Lett..

[44] Schellekens AJ, Van den Brink A, Franken JH, Swagten HJM, Koopmans B (2012). Electric-field control of domain wall motion in perpendicularly magnetized materials. Nat. Commun..

[45] Finizio S (2019). Deterministic field-free Skyrmion nucleation at a nanoengineered injector device. Nano Lett..

[46] Zeissler K (2020). Diameter-independent skyrmion Hall angle observed in chiral magnetic multilayers. Nat. Commun..

[47] Satchell N (2020). Spin-valve Josephson junctions with perpendicular magnetic anisotropy for cryogenic memory. Appl. Phys. Lett..

[48] Bell C (2004). Controllable Josephson current through a pseudospin-valve structure. Appl. Phys. Lett..

[49] Gingrich EC (2016). Controllable 0-$$\pi $$ Josephson junctions containing a ferromagnetic spin valve. Nat. Phys..

[50] Dayton IM (2018). Experimental demonstration of a Josephson magnetic memory cell with a programmable $$\pi $$-junction. IEEE Magn. Lett..

[51] Madden AE, Willard JC, Loloee R, Birge NO (2018). Phase controllable Josephson junctions for cryogenic memory. Supercond. Sci. Technol..

[52] Konc̆ M (1994). Temperature dependence of the magnetization and of the other physical properties of rapidly quenched amorphous CoB alloys. IEEE Trans. Magn..

[53] Schütz G (1990). Spin-dependent X-ray absorption in Co/Pt multilayers and Co$$_{50}$$Pt$$_{50}$$ alloy. J. Appl. Phys..

[54] Geissler J (2001). Pt magnetization profile in a Pt/Co bilayer studied by resonant magnetic X-ray reflectometry. Phys. Rev. B.

[55] Suzuki M (2005). Depth profile of spin and orbital magnetic moments in a subnanometer Pt film on Co.. Phys. Rev. B.

[56] Rowan-Robinson RM (2017). The interfacial nature of proximity-induced magnetism and the Dzyaloshinskii-Moriya interaction at the Pt/Co interface. Sci. Rep..

[57] Inyang O (2019). Threshold interface magnetization required to induce magnetic proximity effect. Phys. Rev. B.

[58] Khasawneh MA, Pratt WP, Birge NO (2009). Josephson junctions with a synthetic antiferromagnetic interlayer. Phys. Rev. B.

[59] Satchell N, Birge NO (2018). Supercurrent in ferromagnetic Josephson junctions with heavy metal interlayers. Phys. Rev. B.

[60] Barone A, Paternò G (1982). Physics and Applications of the Josephson Effect.

[61] Quarterman P (2020). Distortions to the penetration depth and coherence length of superconductor/normal-metal superlattices. Phys. Rev. Mater..

[62] Flokstra MG (2018). Observation of Anomalous Meissner Screening in Cu/Nb and Cu/Nb/Co Thin Films. Phys. Rev. Lett..

[63] Bergeret FS, Volkov AF, Efetov KB (2001). Josephson current in superconductor-ferromagnet structures with a nonhomogeneous magnetization. Phys. Rev. B.

[64] Krupin O (2005). Rashba effect at magnetic metal surfaces. Phys. Rev. B.

[65] Miron IM (2010). Current-driven spin torque induced by the Rashba effect in a ferromagnetic metal layer. Nat. Mater..

[66] Miron IM (2011). Perpendicular switching of a single ferromagnetic layer induced by in-plane current injection. Nature.

[67] Fauré M, Buzdin AI, Golubov AA, Kupriyanov MY (2006). Properties of superconductor/ferromagnet structures with spin-dependent scattering. Phys. Rev. B.

[68] Pugach NG, Kupriyanov MY, Goldobin E, Kleiner R, Koelle D (2011). Superconductor-insulator-ferromagnet-superconductor Josephson junction: from the dirty to the clean limit. Phys. Rev. B.

[69] Bergeret FS, Tokatly IV (2014). Spin-orbit coupling as a source of long-range triplet proximity effect in superconductor-ferromagnet hybrid structures. Phys. Rev. B.

[70] Jeon K-R (2018). Enhanced spin pumping into superconductors provides evidence for superconducting pure spin currents. Nat. Mater..

[71] Herr, A. Y. & Herr, Q. P. Josephson magnetic random access memory system and method (2012). US Patent 8 270 209.

[72] Bourgeois O (2001). Josephson effect through a ferromagnetic layer. Eur. Phys. J. B.

[73] Baek B (2015). Spin-transfer torque switching in nanopillar superconducting-magnetic hybrid Josephson junctions. Phys. Rev. Appl..

[74] The Royce Deposition System is a multi-chamber, multi-technique thin film deposition tool based at the University of Leeds as part of the Henry Royce Institute.

[75] Wang Y, Pratt WP, Birge NO (2012). Area-dependence of spin-triplet supercurrent in ferromagnetic Josephson junctions. Phys. Rev. B.

