# Supplementary material for: Pt and CoB trilayer Josephson π junctions with perpendicular magnetic anisotropy
Source: Sci Rep. 2021 May 27;11:11173. doi: 10.1038/s41598-021-90432-y (PMC8159980; doi:10.1038/s41598-021-90432-y)
Supplement: Supplementary file 1 — Supplementary Information. [file 41598_2021_90432_MOESM1_ESM.pdf]

Supplementary information for: "Pt and CoB trilayer Josephson π junctions with perpendicular magnetic anisotropy"

N. Satchell, T. Mitchell, P. M. Shepley, E. Darwin, B. J. Hickey, and G. Burnell

School of Physics and Astronomy, University of Leeds, Leeds, LS2 9JT, United Kingdom

This supplementary information contains additional analysis and data in support of the conclusions presented in the main text.

S1. DETERMINING MAGNETISATION

The magnetisation data presented in the main text were acquired in a Quantum Design MPMS3 magnetometer. The magnetometer returns measurements of the total magnetic moment of the sample in emu. The total magnetic moment of the sample contains the contributions due to the substrate, thin film, and any spurious signal such as those described by Garcia *et al.* [1] . Careful sample handing was used to minimise spurious contributions to the total moment. The linear diamagnetic background due to the substrate was subtracted from the total measured moment. We report the data after substrate subtraction as the area normalised magnetic moment (moment/area) in emu/cm^2 by measuring the area of the sample.

For a uniform slab of ferromagnetic material, the volume magnetisation (M) can be determined by dividing the saturation moment/area by the thickness of the ferromagnetic layer. M can also be reliably determined by measuring several samples of varying thickness. If one plots moment/area versus thickness (d), as per Figure S1, then the fitted gradient will be M,

$$\text{moment/area} = Md. \tag{S1}$$

In physical systems, a thin ferromagnetic layer is unlikely to be accurately described as a uniform slab. In thin layers the interfaces become significant, so one must account for interfacial contributions to the magnetisation, which may be different than the bulk. Examples of interfacial contributions include magnetic dead layers and the polarization of adjacent layers. Magnetic dead layers can form at interfaces with certain non-ferromagnetic materials or if the surface of the layer becomes oxidised. Of particular relevance to studies of superconductor/ferromagnetc heterostructures is the known magnetic dead layer formation

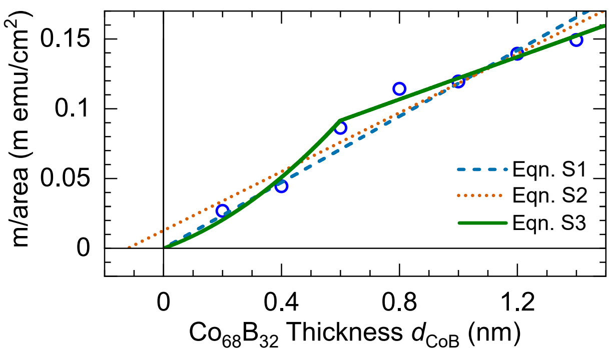

FIG. S1. Magnetic moment/area versus $Co_{68}B_{32}$ thickness data presented in the main text, fit to Equations S1, S2, and S3. The extracted magnetisation of the $Co_{68}B_{32}$ layer from the three fits are $M = 1200 \pm 50$ emu/cm^3 (Equation S1), $M = 1100 \pm 100$ emu/cm^3 (Equation S2), and $M = 760 \pm 90$ emu/cm^3 (Equation S3). Only fitting to Equation S3 returns a magnetisation consistent with the bulk value of 730 emu/cm^3[3].

at Nb/ferromagnet interfaces [2]. Another interfacial effect in thin films is the polarization of the adjacent layer. At some ferromagnet/non-ferromagnet interfaces, the ferromagnetic layer can create a polarization inside the non-ferromagnetic layer by the magnetic proximity effect. Polarization is particularly common at interfaces with Pd and Pt since they are stoner enhanced paramagnets.

To model a magnetic slab with possible magnetic dead layers and/or polarized adjacent layers, one can again plot moment/area versus d and fit to the expression,

$$\text{moment/area} = M(d - d_i), \tag{S2}$$

where d_i is the x-axis intercept. A positive x-axis intercept is indicative of magnetic dead layer formation and the thickness of the dead layer at each interface can be estimated as $d_i/2$. A negative x-axis intercept is indicative that at least one of the adjacent layers has gained a polarization. In this instance, the y-intercept provides the magnitude of the contribution of the polarized layer(s) to the overall measured moment/area.

To describe our Pt/$Co_{68}B_{32}$/Pt data, we first attempt fitting to Equations S1 and S2. The results of these fittings are shown in Figure S1. We find that these fits overestimate the magnetization of our $Co_{68}B_{32}$ layer and underestimate the polarization in the Pt layer. The extracted $M = 1200 \pm 50$ emu/cm^3 (Equation S1) and $M = 1100 \pm 100$ emu/cm^3 (Equation S2) are not consistent with the reported literature value of 730 emu/cm^3 for $Co_{68}B_{32}$ [3]. It

is very well established from the literature that the Pt gains a considerable polarization in thin film multilayers such as ours, which is not accounted for in the fits to Equations S1 and S2 presented in Figure S1.

A simple slab model is, therefore, insufficient to fully describe our Pt/$Co_{68}B_{32}$/Pt system. To fully describe our data on the Pt/$Co_{68}B_{32}$/Pt system presented in the main text we create a toy model based on partial layer coverage. For very thin layers (1 or 2 monolayers), it is possible that the layer coverage is not uniform or that the ferromagnet only partially covers the surface. Such a picture is consistent with common thin-film growth modes, where adatoms initially form islands, which coalesce into complete layer coverage as the film thickness increases. Such incomplete layer coverage will affect the polarization of adjacent layers, as the adjacent layer will only gain polarization in the vicinity of the magnetic islands.

To model the partial layer coverage, we assume that: at zero thickness, the layer coverage is 0%; at a critical thickness, d_{critical}, the islands connect and layer coverage is 100%; layer coverage % increases linearly between those two thicknesses; and at thicknesses above d_{critical}, the data should be described by Equation S2. The equation describing this toy model is,

$$\text{moment/area} = \begin{cases} M(d-d_i)(d/d_{\text{critical}}), & \text{for } 0 < d < d_{\text{critical}} \\ M(d-d_i), & \text{for } d \geq d_{\text{critical}} \end{cases} \tag{S3}$$

The results fitting to this toy model returns a magnetisation $M = 760 \pm 90$ emu/cm^3, which is consistent with the bulk value of 730 emu/cm^3[3]. Equation S3 therefore best represents our Pt/$Co_{68}B_{32}$/Pt system as shown in Figure S1, and so this best fit model is presented in the main text.

S2. TRAPPED FLUX IN SUPERCONDUCTING COILS

The cryostat has a single horizontal field coil. The sample can be rotated about the vertical axis, which we perform in increments of 90° to bring that field in- and out-of-plane of the junctions. In order to avoid trapping flux in the superconducting Nb layers in the devices, we performed all sample rotations in zero field above the T_c of the Nb and always cooled the sample in zero applied field (in practice there will inevitably be a small remanent field due to trapped flux in the magnet). The full sequence of setting the magnetic state of our samples and performing the measurement field sweeps is: warm to 15K, rotate sample

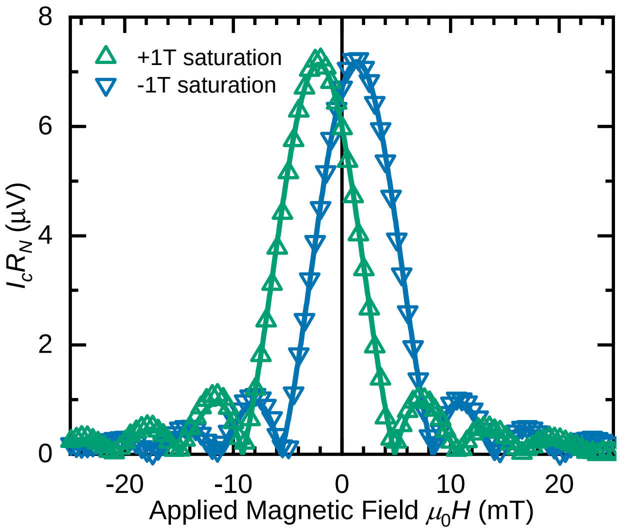

FIG. S2. Product of critical Josephson current times normal-state resistance versus applied magnetic field for ferromagnetic Josephson junctions of the form S-Pt(10)-$Co_{68}B_{32}$(0.6)-Pt(5)-S at 1.8 K. I_c is determined from the measured I–V characteristic at each field value and R_N is the average normal state resistance across all measured fields. The uncertainty in determining I_cR_N is smaller than the data points. The data are fit to Equation 2 and 3 from the main text.

to apply field out-of-plane, apply saturating field, remove saturating field, rotate sample by 90°, cool sample, apply field in-plane, measure. Once we have finished measuring that magnetic state of the sample, we may wish to measure a further condition, such as reversing the magnetisation. To do so, we remove the in-plane field, warm to 15K and repeat the cycle described above.

Figure S2 contains supporting data on the trapped flux in the superconducting coils used in the measurements of our samples. We have observed similar trends by measuring the field directly using a Hall probe during magnetic field sweeps when the sample space was at room temperature. The green data are acquired by sweeping from +25 mT to -25 mT after performing the cycle described above with a +1 T out-of-plane saturating field at 15 K. The blue data are acquired by sweeping from -25 mT to +25 mT after cycling through a -1 T out-of-plane saturating field at 15 K. These data suggest that to compensate for the trapped flux in our 3 T horizontal superconducting Helmholtz coils to achieve zero global applied field requires application of a small opposite field of 1-3 mT.

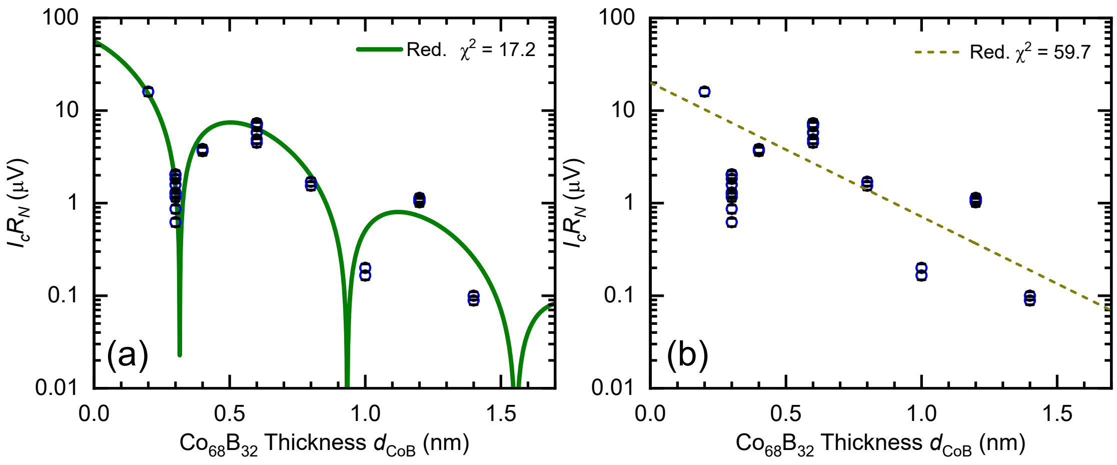

FIG. S3. Product of critical Josephson current times normal-state resistance versus nominal $Co_{68}B_{32}$ thickness for ferromagnetic Josephson junctions of the form S-Pt(10)-$Co_{68}B_{32}$($d_{\rm CoB}$)-Pt(5)-S. Each data point represents one Josephson junction. (a) The best fit of Equation S4 as presented in the main text of our publication, where the reduced $\chi^2 = 17.2$. (b) The best fit of Equation S5, the null hypothesis, where $\xi_F = 0.30$ nm and the reduced $\chi^2 = 59.7$.

S3. GOODNESS OF FIT ANALYSIS

The main conclusion of our work is that the transport properties of Josephson junctions with Pt/$Co_{68}B_{32}$/Pt barriers are consistent with intermediate limit zero-π oscillations. For convenience, we reproduce the equation governing this limit [4],

$$I_cR_N = V_0 \exp\left(\frac{-d_F}{\xi_{F1}}\right)\left|\sin\left(\frac{d_F - d_{\text{zero}-\pi}}{\xi_{F2}}\right)\right|, \tag{S4}$$

where V_0 is the extrapolated I_cR_N at zero thickness, $d_{\text{zero}-\pi}$ is the position of the first zero–π transition, $\xi_{F1} = l_e$ and $\xi_{F2} = \xi_F$ are the lengthscales governing the decay and oscillation of I_cR_N, respectively. For ferromagnetic Josephson junctions where zero-π oscillations are not expected to occur, the decay of supercurrent can be described by the simple exponential decay,

$$I_cR_N = V_0 \exp\big(-d_F/\xi_F\big). \tag{S5}$$

A possible null hypothesis for our reported zero-π oscillations in Pt/$Co_{68}B_{32}$/Pt barriers is if our junctions are best described by Equation S5 and not Equation S4, as this would suggest that zero-π oscillations may not have been observed.

Figure S3 shows goodness of fit analysis of our result compared to the null hypothesis. For

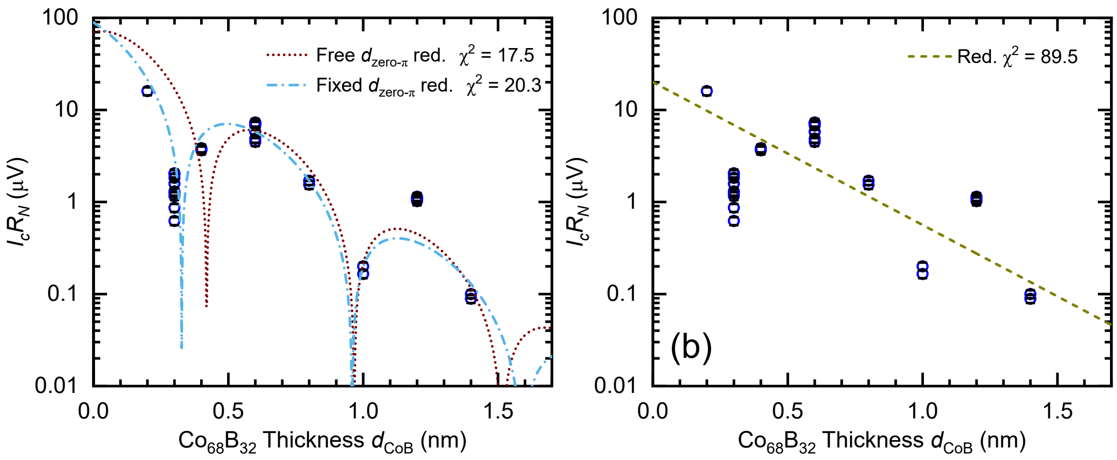

FIG. S4. Product of critical Josephson current times normal-state resistance versus nominal $Co_{68}B_{32}$ thickness for ferromagnetic Josephson junctions of the form S-Pt(10)-$Co_{68}B_{32}$(d_{CoB})-Pt(5)-S. Each data point represents one Josephson junction. In this case, fitting is only performed for the data range $d_{\text{CoB}} \geq 0.6$ nm. (a) Fitting to Equation S4 with either a free or fixed $d_{\text{zero}-\pi}$ parameter. The trends in the fitted parameters over this reduced data range are consistent with fitting over the entire data range. (b) Fit to Equation S5, where $\xi_F = 0.28$ nm and the reduced $\chi^2 = 89.5$.

Figure S3 (a), the best fit of Equation S4 as presented in the main text gives the reduced $\chi^2 = 17.2$. For Figure S3 (b), the best fit of Equation S5 gives the reduced $\chi^2 = 59.7$. Therefore, the χ^2 analysis allows us to reject the null hypothesis that a simple exponential decay can describe our experimental result.

In order to fully describe our magnetisation data, we constructed a toy model of partial layer coverage for samples with the thinnest layers. On the other hand, electrical transport measurements on junctions with the thinnest barriers do not appear to be sensitive to localised partial coverage, as the pair correlations which probe the barrier are averaged over the superconducting coherence length. Nonetheless, we consider next if our findings are robust even when excluding the data from the thinnest barriers.

Figure S4 shows goodness of fit analysis on a reduced data range $d_{\text{CoB}} \geq 0.6$ nm. In Figure S4 (a) we consider the results of two fits to Equation S4. For the first fit attempt, where all parameters in Equation S4 are free parameters (dotted line), the fitting struggles to fit the $d_{\text{zero}-\pi}$ parameter, as the reduced data range does not contain the first zero-π transition. However overall, the trends in the fitted $\xi_{F1} = 0.22 \pm 0.02$ nm and $\xi_{F2} = 0.17 \pm 0.02$ nm are

consistent with our conclusion of intermediate limit transport. Alternatively, we can fix the $d_{\mathrm{zero}-\pi}$ parameter (dot dash line), in which case the best fit returns $\xi_{F1} = 0.22 \pm 0.02$ nm and $\xi_{F2} = 0.20 \pm 0.01$ nm. By eye, this fit is a plausible explanation of our results over the entire data range. The returned ξ_{F2} values from these two fitting methodologies, where ξ_{F2} governs the period of the zero-π oscillation (the key result of our work), are consistent, within error, with ξ_{F2} by fitting over the entire data range (0.20 ± 0.02 nm), providing us confidence in our reported result. In Figure S4 (b) we fit to the simple exponential decay Equation S5, over the reduced data range, and find $\xi_F = 0.28$ nm and the reduced $\chi^2 = 89.5$. The χ^2 analysis allows us to reject the null hypothesis that a simple exponential decay can describe our experimental result, even over the reduced data range $d_{\mathrm{CoB}} \geq 0.6$ nm.

S4. POSSIBLE SPIN-TRIPLET COMPONENT OF SUPERCURRENT

Josephson junctions containing a source of s-wave superconductivity, large spin-orbit coupling, and ferromagnetism are predicted to display transport properties consistent with spin-triplet supercurrents [5]. Assuming that the total supercurrent in the junction is the sum of the spin-singlet and spin-triplet components, we can describe the total supercurrent as;

$$I_c R_N = V_0 \exp\left(\frac{-d_F}{\xi_{F1}}\right)\left|\sin\left(\frac{d_F - d_{\mathrm{zero}-\pi}}{\xi_{F2}}\right)\right| + V_{\mathrm{Triplet}} \exp\left(\frac{-d_F}{\xi_{\mathrm{Triplet}}}\right), \tag{S6}$$

where V_{Triplet} and ξ_{Triplet} are the critical voltage and decay length of the spin-triplet component of supercurrent, respectively.

To determine if spin-triplet supercurrents contribute to our transport results, we fit Equation S6 to our junction data, shown in Figure S5. We find that $V_{\mathrm{Triplet}} \rightarrow 0$, and the other fit parameters correspond to those of the singlet only fit described in the main text. This suggests that the data are well described by singlet transport physics alone and any spin-triplet component is below our experimental sensitivity.

An alternative methodology is to consider whether we might be able to observe a spin-triplet supercurrent of the magnitude reported in literature. Figure S5 shows simulations of Equation S6 with a weak spin-triplet component coexisting in our junctions, inspired by the findings of Glick *et al.* for a PMA synthetic antiferromagnet system with intentional spin-mixing layers [6]. Parameter set 'Triplet 1' corresponds to a decay length of spin-triplet component being only twice that of the spin-singlet component and $V_{\mathrm{Triplet}} = \frac{1}{7}V_0$. These

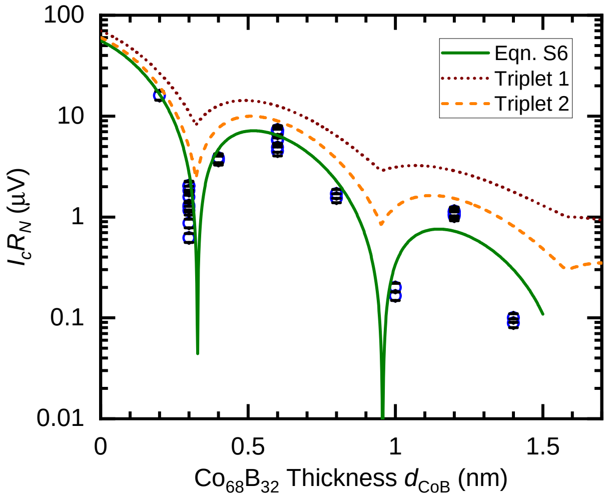

FIG. S5. Product of critical Josephson current times normal-state resistance versus nominal $Co_{68}B_{32}$ thickness for ferromagnetic Josephson junctions of the form S-Pt(10)-$Co_{68}B_{32}(d_{CoB})$-Pt(5)-S. Each data point represents one Josephson junction. The solid green curve shows the best fit of Equation S6, which recovers the fit presented in the main text of our publication as $V_{\mathrm{Triplet}} \to 0$. The dotted and dashed lines correspond to the simulations of Equation S6 to two parameter sets described in the text

parameters correspond to the results of Glick *et al.* with symmetric Ni spin-mixer layers. Parameter set 'Triplet 2' corresponds to a decay length of spin-triplet component being twice that of the spin-singlet component and $V_{\mathrm{Triplet}} = \frac{1}{21}V_0$. These parameters correspond to asymmetric Ni and NiFe spin-mixer layers. These simulations suggest that a spin-triplet supercurrent of the magnitude reported in PMA synthetic antiferromagnets by Glick *et al.* should be observable as the major suppression of the oscillations and higher I_cR_N in the thicker samples in this work.

[1] M. A. Garcia, E. Fernandez Pinel, J. de la Venta, A. Quesada, V. Bouzas, J. F. Fernández, J. J. Romero, M. S. Martín González, and J. L. Costa-Krämer, J. Appl. Phys. **105**, 013925 (2009).

[2] J. W. A. Robinson, S. Piano, G. Burnell, C. Bell, and M. G. Blamire, Phys. Rev. B **76**, 094522 (2007).

[3] M. Konč, P. Spišák, P. Kollár, P. Sovák, O. Duša, and T. Reininger, IEEE Trans. Magn. **30**, 524 (1994).

[4] F. S. Bergeret, A. F. Volkov, and K. B. Efetov, Phys. Rev. B **64**, 134506 (2001).

[5] F. S. Bergeret and I. V. Tokatly, Phys. Rev. B **89**, 134517 (2014).

[6] J. A. Glick, S. Edwards, D. Korucu, V. Aguilar, B. M. Niedzielski, R. Loloee, W. P. Pratt, N. O. Birge, P. G. Kotula, and N. Missert, Phys. Rev. B **96**, 224515 (2017).
